# Challenges in Paediatric Laparoscopic Surgeries

**Published:** 2009-10

**Authors:** Ruchi Gupta, Saru Singh

**Affiliations:** 1 Prof & Head, Deptt. of Anaesthesia, Sri Guru Ram Das Institute of Medical Sciences & Research, Amritsar – 143001, Punjab; 2 Assistant Professor, Deptt. of Anaesthesia, Sri Guru Ram Das Institute of Medical Sciences & Research, Amritsar – 143001, Punjab

**Keywords:** Paediatric Laparoscopy, Anaesthetic challenges

## Abstract

**Summary:**

Today in the era of minimally invasive surgery, paediatric laparoscopy has become widely popular.The anaesthetic management in these cases poses special problems due to pneumoperitoneum created and extremes of position adopted in addition to the fact that paediatric anaesthesia itself is a challenge. Mostly the physiological as well as anaesthetic consideration are same except that child is not a small adult. The pressure of pnemoperitoneum needs to be kept between 6-12cm H_2_O, flow of gas about 0.9l, ventilation to be controlled, temperature monitoring being essential, use of atropine as premedicant, intravenous fluid management to be meticulous, induction with sevoflurane preferred as children may not allow i.v.puncture, intraoperative surgical complications being more, one needs to be very vigilant to diagnose and treat it. Using periumbilical area in paediatric age group should be avoided because the umbilical vessels have not involuted and can get punctured.Thus careful management in paediatric laparoscopic surgery will assume an important place in paediatric surgery.

## Introduction

Paediatric laparoscopy has been first described in 1923 by Kelling but its use has increased since last decade. A laparoscopic approach offers several advantages over an open procedures; potentially reduces the surgical stress and fluid shifts that may accompany it; in addition there is less need for postoperative analgesia, reduction of postoperative respiratory and wound complications; shortens postoperative convalescence, including an intensive care unit stay; rapid return to normal diet and decreased overall hospital stay.^1^ Laparoscopic procedures in children can be done for number of indications (Table 1) and anaesthesia for these procedures poses certain challenges which increase manifolds as the patients are children.

**Table 1:** Indications of laparoscopic surgeries in children

**Diagnostic** **Operative** Adrenalectomy Appendicectomy Cholecystectomy Diagphragmatic hernia repair Fundoplication Gastrostomy Herniorraphy Intestinal procedures Nephrectomy Orchidopexy Orchidectomy Urological procedures

## Physiological Considerations:

**Fig 1:**
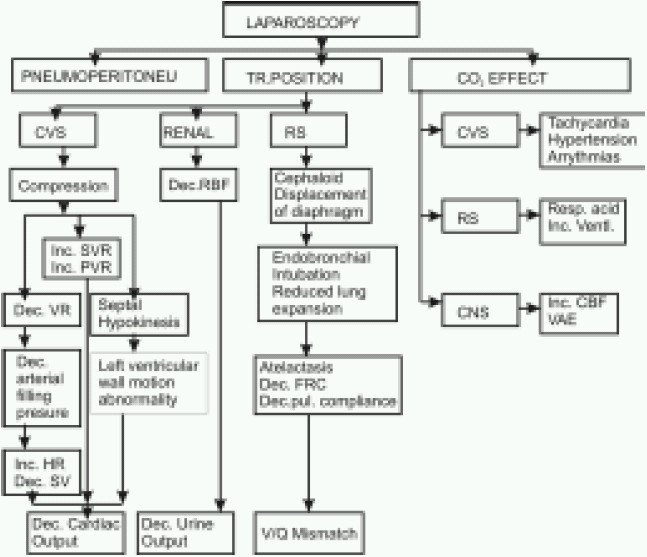
Physiological effects of laparoscopy

Physiologic changes during laparoscopic surgery in children is almost similar to adults. What makes anaesthesia challenging in these cases are related to positioning (Trendelenburg, reverse Trendelenburg) and creation of pneumoperitoneum. The magnitude of these changes is influenced by the patient's age, underlying myocardial function and anaesthetic agents.^2^ (Fig 1)

CVS-cardiovascular system, CNS-central nervous system, RS-respiratory system, TR-trendelenburg, VR-venous return, PVR-pulmonary vascular resistance, SVR-systemic vascular resistance, SV-Stroke volume, HR-heart rate, V/Q-ventilation perfusion, FRC– functional residual capacity, CO2-carbondioxide, Resp.Acid-Respiratory Acidosis, Ventl-Ventilation, RBF-Renal blood flow,CBF-cerebral blood flow, VAE-Venous air embolism, Dec-Decrease, Inc-Increase.

The gases that can be insufflated are CO_2_, N_2_O, air and helium. Out of these CO_2_ is the most commonly used gas because of its inability to support combustion, rapidly excreted, high blood solublity and minimal effects of its intravascular embolisation. The creation of pneumoperitoneum with insufflated gas permits better visualization and manipulation of the abdominal viscera. The volume of insufflating gas necessary for pneumoperitoneum is much lower in children than adults. Adults require 2.5L to 5L where as a 10 kg patient needs about 0.9L.^3^

## Cardiovascular system:

Paediatric laparoscopic procedures are likely to cause an increase in pulmonary and systemic vascular resistance, sudden bradycardia during pneumoperitoneum because of raised intraabdominal pressure(IAP), the chances being much more than adults. Children have a high level of vagal tone and occasionally peritoneal stimulation by a blast of insufflated gas or penetration by trocars and laparoscopes can provoke bradycardia or asystole.^3^,^4^ Intra Abdominal Pressure(IAP) is a critical determinant of cardiovascular stability during laparoscopy.Various cardiovascular effects have been shown in Table 2.

**Table 2:** Cardiovascular effects during laparoscopy in patients without cardiac disease

Vasovagal reflex response to peritoneal stimulation from trocar or insufflations Myocardial sensitization by halothane Reduced venous return Hypovolemia Hypercapnia Venous gas embolism

Patients with normal cardiovascular function tolerate variations in preload and afterload well, but those with cardiovascular disorders, anemia or hypovolemia require meticulous attention to volume loading, positioning and insufflation pressures. For this restriction of the IAP to 6mmhg in infants and to 12mmHg in older children has been recommended. These pressures have minimal effects on cardiac index.^6^,^7^

## Respiratory System

Various pulmonary effects include reduced diaphragmatic excursion & cephalad shift; reduced thoracic compliance & functional residual capacity; early closure of smaller airways & increased peak airway pressures; thus leading to ventilation perfusion mismatch. Other problems occuring during laparoscopy include endobronchial intubation, subcutaneous emphysema, pnuemothorax, pneumomediastinum, massive CO_2_ embolism, bronchospasm, bronchial hypersecretion and atelectasis.^8^

These pulmonary effects depend on age, weight, preoperative pulmonary functions, degree of trendelenburg position, anaesthetic agents, and ventilation technique. In order to reduce these effects neuromuscular blockade, endotracheal intubation, adjustment in mechanical ventilation (PCV) and PEEP are recommended.^2^

## Central Nervous System:

Hypercapnia, high systemic vascular resistance and head low position combine to elevate intracranial pressure.^7^ The induction of pneumoperitoneum itself increases middle cerebral artery blood flow velocity in young children, even when CO_2_ reactivity is normal.^9^ Avoiding above factors may help reduce intracranial pressure.

## Gartrointestinal System:

Increased intraabdominal pressure increases the risk of regurgitation. This is more so if regional anaesthesia with sedation or GA with mask ventilation is given.^10^ So the use of acid aspiration prophylaxis has been recommended in all patients.

## Coagulation System:

Increased IAP may lead to increased venous stasis thus causing deep vein thrombosis especially when surgery is prolonged,^11^ so deep vein thrombosis prophylaxis should be done in such patients.

## Metabolic

and acute phase responses (glucose, leucocytosis, C-reactive protein) and interleukin were less after laparoscopy compared to laparotomy.^1^,^12^ But reduction in splanchnic, hepatic, and renal blood flow and increase in the plasma concentrations of catecholamines, cortisol, insulin, epinephrine, prolactin, growth hormone, and glucose levels have been reported with carbon dioxide pneumoperitoneum.^2^

## Surgical Considerations

Laparoscopic surgery involves the intraperitoneal or extraperitoneal insufflation of carbon dioxide through a veress needle. A variable-flow insufflator terminates flow at a preset intra-abdominal pressure which is decided according to the age group of patients. Once the abdomen is filled with carbon dioxide, the veress needle is replaced by a cannula through which a video laparoscope is inserted. Additional ports are placed according to the surgical procedure undertaken.^2^ In infants less than 5 kg weight, periumbilical area should not be used for port access because of risk of puncture of umbilical vessels.^10^After tracheal intubation, the stomach is suctioned with an orogastric tube and bladder catheterization is done in order to decrease the risk of visceral injury during trocar insertion.

## Anaesthetic Considerations

Although regional anaesthesia has been used in adults & older children, general anaesthesia is nearly always used for laparoscopic procedures in infants & children.^2^

## Preoperative Evaluation

Detailed history and physical examination should be conducted in all elective as well as emergency procedures. Investigations should be ordered according to the requirement of the procedure(type, urgency) and co existing diseases..7

## Premedication

Children younger than 9 months do not suffer separation anxietyand require no sedatives however older children may be given oral midazolam 0.5–0.75mg.kg^-1^ 15–30 min preoperatively. Other drugs include antacids, H_2_ antagonists, gastrokinetic agents, opioids and ketamine. Anticholinergic (atropine 20µg.kg^-1^ i.m.) is associated with decreased incidence of cardiovascular and airway complications perioperatively. It also helps in preventing vasovagal reflexs occurring during peritoneal penetration..7

## Induction

Venous access can be performed with minimal discomfort using EMLA, prilocaine gel and should be secured above the level of diaphragm, so as to permit rapid fluid resuscitation in the case of accidental vascular injury. A fluid bolus of 20 ml.kg^-1^ can be used to offset hemodynamic effects when pnuemoperitoneum is created.^7^ Induction of GA can be done in children by inhalational agents (sevoflurane or halothane, air and oxygen) or by intravenous route if venous access has already been secured. Tracheal intubation which is a gold standard in children, provides secure airway, allows good muscle relaxation, optimal surgical conditions and controlled ventilation.^2^ The laryngeal mask airways(LMA) or mask ventilation for airway maintenance for short-term laparoscopic urologic procedure has been used. For inguinal herniorraphy, orcheopexy, or orchiectomy; it is a suitable and safe alternative to endotracheal intubation.^13^ Mask ventilaton is beneficial in asthmatic patients where intubation needs to be avoided. In case of pulmonary disease, spontaneous ventilation using mask has the added advantage of decreasing ventilation perfusion mismatch.^7^

However the role of LMA in upper abdominal procedures is limited due to increased chances of regurgitation. More favorable ventilation and a reduction in inadvertent gastric insufflation have been reported with the LMA-ProSeal.^7^

## Maintenance of Anaesthesia

Anaesthesia is maintained with a volatile agent in oxygen and air. Both isoflurane and halothane have been used successfully.^7^ However, there is increased chances of arrhythmias occurring with halothane in spontaneously breathing patients due to hypercarbia.^3^Moreover there is a risk of halothane hepatotoxicity due to decreased hepatic blood flow.^7^ Isoflurane has been associated with excessive secretions and bronchospasm.^13^Total intravenous anaesthesia (TIVA) using a propofol infusion has also been successfully employed.Minute ventilation needs to be increased by 20% or more to maintain normocapnia.^6^ Nitrous oxide is generally not used as it can increase bowel distension, nausea and vomiting.

## Intraoperative Analgesia

Intraoperative pain management can be done by parenteral, spinal, epidural and caudal route using local anaesthetics, NSAIDS & opioids and this can continue in postoperative period.

## Temperature maintenance

Small children have a high body surface area to mass ratio and little subcutaneous fat or body hair to retain heat. Continuous insufflation of large volumes of cold, non-humidified CO2 directly in to the abdominal cavity also contributes to a major risk of hypothermia. A warming mattress, heated humidifier or a convective forced air warmer might be used if available. Hypothermia is avoided by warming the insufflating gas and/or maintaining insufflating flows of less than 2 L/min.^2^

## Monitoring

Apart from the mandatory monitoring which includes pulse oximetry, NIBP, ECG, capnography and temperature; precordial or oesophageal stethoscope should be used especially for early detection of endobronchial intubation.^6^,^15^ Precordial doppler probe and transoesophageal echocardiography have been recommended in normal patients for the detection of gas embolism and assessment of preload & cardiac contractility in children with cardiac disease.^3^,^16^,^17^ CVP may be indicated where there are more chances of embolism as embolised gas can be aspirated.

## Positioning

The table position itself may need to be changed repeatedly during the operation; both the trendelenburg and the reverse trendelenburg positions are often used. Infants may be placed near the foot end of the table. Accordingly, care must be taken to secure the patient to the table (e.g., using rolls of gauze and tape). Well padding of extremities should be ensured.^2^

## Reversal / Extubation

Reversal and extubation is done after ensuring adequate orogastric suction and empting of pneumoperitonem. Bilateral air entry should be checked at the end of anaesthesia.

## Post operative

## Nausea Vomiting (PONV):

The incidence of PONV following laparoscopy can be reduced by administrating a combination of drugs including ondansetron(100µg.kg^-1^), dexamethsone (150µg.kg^-1^), droperidol(25µg.kg^-1^). Other maneuvers that prevent post operative nausea vomiting are; use of propofol as an induction agent and avoidance of nitrous oxide.

## Respiratory changes:

Additional CO_2_ load can persist into the post operative period resulting in increase in the ventilatory requirement, especially when the ability to increase ventilation is impaired by residual anaesthetic drugs, diaphragmatic dysfunction and parenteral narcotics.^3^

## Pain

Pain following laparoscopy is due to rapid distension of peritoneum, visceral manipulation, irritation and traction of vessels and phrenic nerves, presence of residual gas and inflamatory mediators.^2^,^18^ Normal presentation in adults is abdominal discomfort and shoulder tip pain but in children shoulder tip pain is less.

Pain can be controlled using a multimodal approach such as local anaesthetics, opioids, NSAIDS and adjuncts like clonidine. This can be achieved by instilling local anaesthetic with laparoscope, use of caudal and epidural catheter and bilateral rectus sheath block.^7^ Analgesia can be given as fentanyl 2–5 mcg.kg^-1^ bolus, followed by infusion at 2 mcg.kg^-1^.hour^-1^; or codeine phosphate 1–2 mg.kg^−1^ intramuscularly given at the end of the procedure, or an infusion of remifentanil 0.1–1 mcg.kg^-1^.min^-1^ followed by morphine 0.1–0.2 mg.kg^-1^. At the end of surgery, paracetamol 15–20 mg.kg^-1^ or diclofenac 1–2 mg.kg^-1^, if not contraindicated, can be administered rectally

## Special Considerations

### Diagnostic Laparoscopy in Portal Hypertensive Children(PHT)

The PaCO_2_ increased remarkably in children with PHT undergoing laparoscopy. Absorption of CO_2_ is enhanced by enlarged and tortuous collateral vessels over parietal and visceral peritoneal surfaces, angiomatous channels over liver, increased plasma volume, hyperdynamic circulation and increased cardiac output. Moreover, pulmonary dysfunction and difficulty in ventilation due to enlarged spleen or liver explain the high PaCO_2_ and significantly low PaO_2_. Limiting the duration of CO_2_ pneumoperitoneum and intraabdominal pressure, application of high FiO2 (50%) and adjustment of ventilatory variables is important in such cases.^19^

### Laparoscopic Adrenalectomy for Pheochromocytoma in a Pediatric Patient

Critical events in laparoscopic adrenlectomy occur during intubation, induction of pneumoperitoneum and tumor manipulation. The intravenous infusions of sufentanil, esmolol, and nicardipine should be carefully titrated to attenuate the wide fluctuations in hemodynamics.^9^

MgSO_4_ has been used as an adjunct to nicardipine in pediatric laparoscpoic adrenalectomy which acts by inhibiting the release of catecholamines, blocks catecholamine receptors directly, dilating action on vessel walls and has an antiarrhythmic effect on the heart.^1^,^20^

### Laparoscopic Nissen's Fundoplicaton

Patients undergoing laparoscopic Nissen's fundoplicaton have preexisting respiratory disease secondary to chronic aspiration; and interference with diaphragmatic function during fundoplication lead to post operative hypoxemia. At high IAP levels dissection of esophageal hiatus may even permit the passage of insufflated gas across the diaphragm leading occasionally to pneumothorax and pnemomediastinum. This necessitates routine post operative chest X-ray. These patients may develop hypotension and bradycardia during reverse trendelenburg position; volume loading and administration of vagolytic drugs helps preventing this problem.^21^

## Laparoscopic Renal Surgery in Infants

In a study on 17 patients less than 10 kg weight, laparoscopic renal surgery (nephrectomy, partial nephrectomy, nephroureterectomy) was done. All operations had minimal estimated blood loss, less operative time, reduced hospital stay, low morbidity and rapid recovery. In these patients diaphragmatic injury is common which needs immediate diagnosis and repair.^22^

## Recent Advances:

A new technique known as gasless laparoscopy eliminates the risks of pneumoperitoneum by using mechanical retraction and is therefore appealing for patients with severe heart and pulmonary disease.^5^ Reduced visualization is associated with this technique and its application to pediatrics remains uncertain.

## Challenges

- Preoperative optimization of patients with co existing diseases (eg.PHT, gastrooesophageal reflux, pheochromocytoma) and those posted for emergency surgery.
- Intraoperative diagnosis and treatment of effects of carboperitoneum; maintenance of IAP between 6-12 mmHg. Vigilant observation of its effects and tailoring the management accordingly is the key to successful management.
- Monitoring ECG, NIBP, Pulse oximetry, Capnography, PNS and Temperature for the early detection of hypotension, bradycardia, arrythmias, venous air embolism, endobrochial intubation, pneumothorax and hypothermia is mandatory.
- Post operative care for observation and management of nausea vomiting, inadequacy of ventilation due to residual carbon dioxide load and pain which includes cautious selection of drugs and their routes so as to avoid enhancement of above problems.
